# Exploring the hidden curriculum in public health: a qualitative study

**DOI:** 10.3389/fpubh.2026.1945036

**Published:** 2026-10-22

**Authors:** Woon Chien Cecilia Teng, Wei Ern Ivan Ng, Pei Xuan Annabeth Lee, Shih Yun Faith Nicole Tan, Raymond Boon Tar Lim

**Affiliations:** 1 Saw Swee Hock School of Public Health, National University of Singapore, Singapore, Singapore; 2 National University Health System, Singapore, Singapore

**Keywords:** Asia, hidden curriculum, internships, public health, qualitative, written reflections

## Abstract

**Background:**

The hidden curriculum refers to the subtle ways that knowledge and behavior are learnt outside formal teaching contexts. Widely studied in clinical healthcare, it remains underexplored in public health. As the scope and function of public health practice differ substantially from clinical practice, the hidden curriculum in public health may be distinct from that in clinical fields. We explored the hidden curriculum in public health through undergraduate interns' experiences.

**Methods:**

Using a qualitative design, we examined how public health students made sense of their internship experiences in written reflections. The reflection prompts were informed by Gaufberg et al.'s (2010) definition of the hidden curriculum. Reflexive thematic analysis using a deductive-inductive approach was applied on 36 reflection scripts collected between 2024 and 2025.

**Results:**

We developed three themes (stakeholder engagement as an art, effecting change takes time, and developing effectiveness as a public health professional) comprising ten sub-themes. Positive aspects of the hidden curriculum comprised learning the art of stakeholder engagement, change agency despite constraints, and organizational structure as a necessity, while negative ones included the perceived role of public health in clinical settings, organizational influence on behavior, and mentoring as invalidating. Findings elucidate undercurrents of power and hierarchy in public health practice.

**Conclusions:**

A multi-ethnic Asian student population extended the hidden curriculum literature beyond its predominantly Western base. Future research incorporating larger, more diverse samples, practitioners' perspectives, and longitudinal designs is needed to more fully characterize the hidden curriculum in public health and strengthen professional training.

## 1 Introduction

Traditionally a subspeciality of medicine, public health has since evolved into a distinct field that draws on both medical and non-medical expertise (1–3), with a varied range of practice areas such as global health, health promotion, health systems and policy, social and behavioral sciences (4). As the multidisciplinary value of public health gained wider recognition from the early twentieth century, higher education institutions began to offer public health education to non-medical students (initially at the postgraduate level, and subsequently at the undergraduate level) and establish dedicated public health schools to meet the need for a diverse public health workforce (1, 2, 5, 6). The plurality of professional backgrounds among both trainees and practitioners of public health today (7) suggests that the hidden curriculum in public health may differ substantially from that of clinical disciplines.

The hidden curriculum has been widely recognized as a powerful socializing force in professional development. Definitions of the hidden curriculum, however, vary considerably across the literature (8, 9). Jackson (10) described it as the tacit ways in which knowledge and behavior are constructed outside formal course materials and lessons (8). Gaufberg et al. (11) defined it as “learning that occurs by means of informal interactions among students, faculty, and others and/or learning that occurs through organizational, structural, and cultural influences intrinsic to training institutions”. Hafferty (12) employed a tripartite framework, distinguishing the hidden curriculum (understood as a function of “organizational structure and culture”) from the formal (or official) curriculum and informal curriculum (described as a spontaneous, interpersonal mode of teaching and learning). Shaped by broader societal forces, the hidden curriculum can in turn influence society as students transition into the workforce (13).

Historically, hidden curriculum studies tended to focus on its negative dimensions. The phenomenon has even been regarded as one that is “middle-class, male, and white-dominated” (14) and socially controlling (15, 16). This critical orientation was also reflected in medical education literature (8), following Hafferty's (12) introduction of the concept to the field. Nevertheless, the hidden curriculum's positive effects remain relatively underexplored, with a more balanced approach applied less consistently in the literature (8, 17–19).

Some negative effects of the hidden curriculum include unprofessional and unethical behaviors by superiors or co-workers (11, 20, 21), emotional suppression (11, 21), physician burnout and skepticism (22), and negative stereotyping (21, 23), while positive influences include moral and ethical growth (17), the fostering of trust-based physician-patient relationships (18), and developing the capability to navigate ambiguity (19). The hidden curriculum in other healthcare disciplines (except nursing) is less well-studied, although a similar pattern of findings has been reported (9, 24–27).

Documented evidence on the hidden curriculum in public health is, however, markedly limited. Calos and Pallai (28) argued how professionalism standards in public health reinforce the very bias and inequities that public health seeks to address. Likewise, the hidden curriculum experiences of minoritised students in a London-based health and social care degree programme reflected a colonized curriculum and the pervasive effects of systemic racism (13). Anderson et al. (29) proposed key areas of the hidden curriculum in North American global health education warranting more in-depth exploration, including the field's implicit ethnocentric approaches, and the perpetuation of a “hero myth” among students. Analyses of health promotion and socio-behavioral textbooks highlighted a disproportionate emphasis on individual-level behavioral factors over social determinants of health, the prioritization of theory in understanding and modifying behavior, and insufficient attention to upstream determinants (30, 31).

The available evidence on the hidden curriculum in public health is, however, either opinion-based (28, 29), or, where empirical, confined to critical discourse analyses of selected textbooks (30, 31) or classroom contexts (13), failing to capture the full scope of the public health training experience. Further, the existing literature is predominantly rooted in North American, British, and Dutch contexts. As a sociological construct shaped by cultural and institutional norms, the hidden curriculum's operation and effects may differ meaningfully across settings. To date, no primary studies have investigated the hidden curriculum from the perspective of non-medical public health trainees in workplace settings. A more comprehensive understanding of how it shapes the public health training experience is therefore needed to support the deliberate cultivation of professional identities and values among a workforce defined by its disciplinary diversity. We sought to address these identified gaps in this study, which aimed to explore the hidden curriculum in public health through the lens of public health interns.

## 2 Methods

A qualitative methodology was employed to explore the hidden curriculum in public health by examining how public health interns made sense of their internship experiences in written reflections. We adopted a constructionist approach, treating these reflections as accounts influenced by institutional context. The study was reviewed and approved by the Departmental Ethics Review Committee (Protocol #208).

### 2.1 Learning environment

This study was conducted between 2024 and 2025 in an undergraduate public health internship course, offered annually as minor elective or second major capstone, at the only national school of public health within a large research-intensive university in Singapore. Students undertake internships between May to July at public, private, and non-profit organizations involved in public health work (including government agencies, healthcare institutions, global health organizations, medical consultancies, social service agencies, and academia) supervised by public health practitioners (“supervisors”). Most internship arrangements were fully on-site or part-remote.

Enrolment requires completion of at least two core and two elective public health minor courses. Enrolled students must fulfill a minimum of 320 h (8 weeks) on a full-time basis. Grading is satisfactory/unsatisfactory, based on internship workshop attendance, supervisor evaluations, and internship training records. Workshops are conducted with the institution's career advisory unit, and cover internship readiness and personal branding.

Singapore's higher education culture is often characterized by a strong emphasis on academic achievement and hierarchical student-teacher relationships (32), which may shape how students navigate the hidden curriculum (including patterns of help-seeking and deference to senior figures) differently from Western contexts, from which most hidden curriculum literature originates.

### 2.2 Data collection

At the start of the internship, all enrolled students received two reflection prompts as part of formal instruction: (i) a challenge faced and how they overcame it (500 words, mid-internship), and (ii) the hidden curriculum observed during the internship (1,500 words, end-internship) (Supplementary Materials). Reflections were not graded.

To capture the multiple ways implicit learning manifests in workplace settings such as internships, the second prompt was adapted from Gaufberg et al.'s (11) broader conceptual definition of the hidden curriculum. Students were encouraged to write honestly and not “for the teacher”. Recruitment was conducted by a research analyst with no teacher-student relationship with participants. All students from both cohorts were invited to participate in the research, and informed that participation was voluntary and would not affect their course result. Written reflections were contributed for analysis after obtaining written informed consent. Reflection scripts were de-identified and imported into Taguette 1.4.1 (33) for coding.

### 2.3 Data analysis

Reflexive thematic analysis (34) was conducted using a deductive-inductive approach, with deductive codes informed by Gaufberg et al.'s (11) conceptualization of the hidden curriculum as outlined in the Introduction, and inductive codes generated from the data. In keeping with the study aims, both the hidden curriculum's negative and positive effects were explored. The first three authors familiarized themselves with the data through repeated reading, after which the second and third authors generated initial codes. Coding was piloted on a subset of scripts to develop a preliminary codebook. This codebook was iteratively refined through discussion among the first three authors and applied to the remaining scripts, with codes organized into candidate themes and sub-themes. These were further developed and refined through discussions among all five authors, focusing on both semantic and latent features within the data. Discrepancies at each stage were resolved through discussion. Representative excerpts were selected from the data to illustrate each sub-theme.

### 2.4 Researcher reflexivity

The research team comprised of junior and senior researchers. The second and third authors are junior researchers, students reading a public health minor or second major, and they contributed the student perspective which shed valuable insights into the experiences of public health interns. They were trained in thematic analysis under the supervision of the first, fourth, and last authors who are senior researchers. The fourth author is a research analyst. The first and last authors are faculty members with experiences ranging from preventive medicine, medical and pharmacy practice, public health education and research. These relevant backgrounds and experiences in the team allowed for the data to be interpreted meaningfully from multiple perspectives.

## 3 Results

### 3.1 Student characteristics

The student demographics from the 2024 and 2025 intakes are shown in Table 1. Most students were female, Singaporean Chinese, in their third or fourth year of study, and reading a primary major in life sciences. More than half of the students were enrolled in a public health second major programme. Among the 39 students enrolled in the course, 18 (46%) students provided written consent to the use of their reflections for the study, resulting in a total of 36 reflection scripts (two reflections per student).

**Table 1:** Demographics of enrolled students (proportions in brackets).

Intake	2024	2025
**Total enrolment**	15	24
**Female**	12 (0.80)	15 (0.60)
**Students in 3rd or 4th year**	12 (0.80)	12 (0.50)
Ethnicity		
Chinese	10 (0.67)	15 (0.63)
Malay	2 (0.13)	4 (0.17)
Indian	2 (0.13)	3 (0.13)
Others	1 (0.07)	2 (0.08)
Public Health programme enrolled in		
Minor	7 (0.47)	6 (0.25)
Second major	8 (0.53)	18 (0.75)
Primary major		
Biomedical engineering	2 (0.13)	2 (0.08)
Business	1 (0.07)	0
Computer science	0	1 (0.04)
Data science	1 (0.07)	0
Data science and economics	0	1 (0.04)
Economics	0	1 (0.04)
Life sciences	9 (0.60)	17 (0.71)
Political Science	0	1 (0.04)
Sociology	2 (0.13)	1 (0.04)

Percentages may not add up to 100% due to rounding.

### 3.2 The hidden curriculum in public health

Three themes and ten (level 1) subthemes were developed (Table 2, Figure 1; illustrative quotes in Supplementary Materials). These are described in the following sub-sections.

**Table 2:** Themes and subthemes.

Themes	Level 1 subthemes	Level 2 subthemes	Level 3 subthemes
Stakeholder engagement as an art	Collaborative dynamics	Working through differences	
		Mentoring	Mentoring as empowering
			Mentoring as invalidating
			Help-seeking as a balancing act
		Competing priorities in clinical settings	
	Person-centric communication	Framing	
		Cultural competence	
		Humanism	
Effecting real change takes time	Organizational structure as a determinant of change	Impedes meaningful change	Structural inefficiencies and constraints
			Influence on workplace behavior and attitudes
			Implementing incremental changes over novel solutions
		A necessity	
	Broader-level barriers		
	Recipient-level barriers		
	Agency despite constraints		
Developing personal effectiveness as a public health professional	Building resilience		Self-leadership
			Growth mindset
	Managing personal-work values incongruence		
	Self-evaluation of career fit		
	Broadening of worldview		

**Figure 1:**
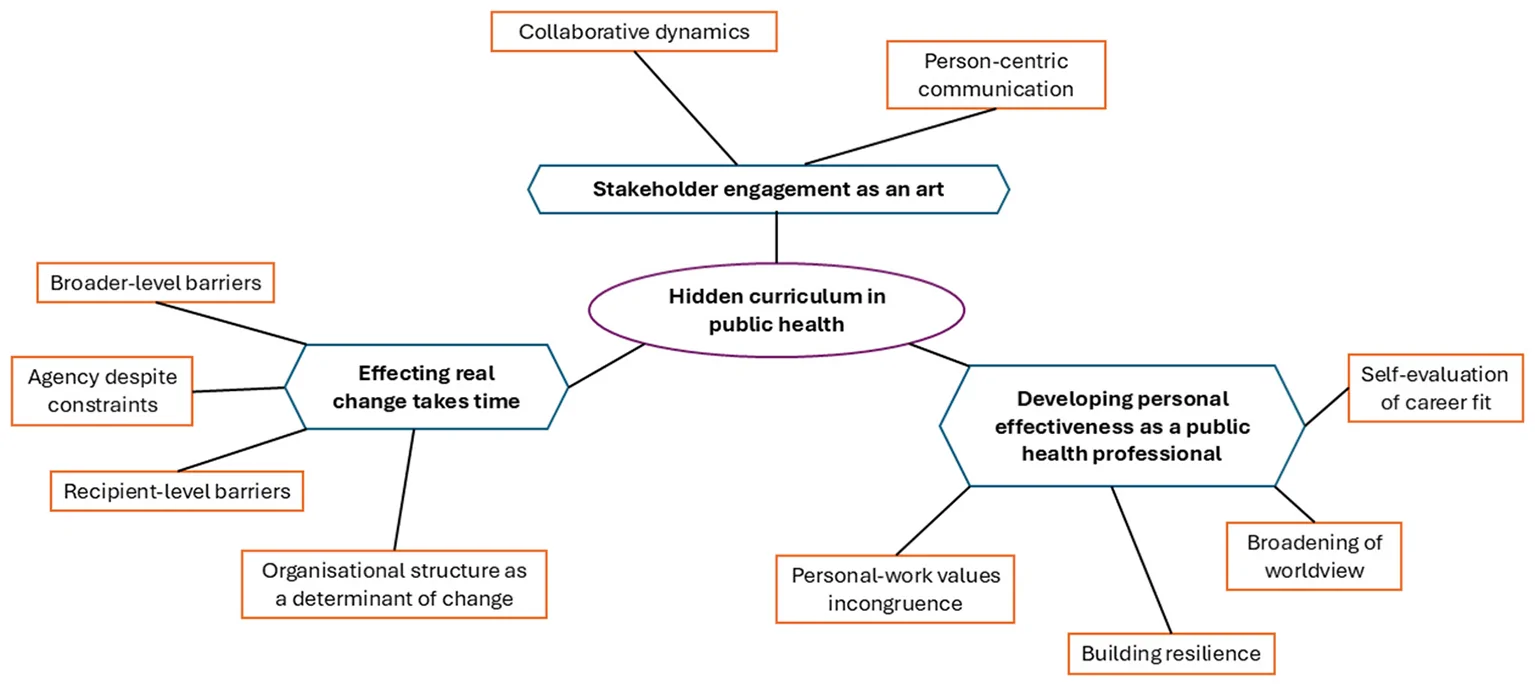
Thematic map.

#### 3.2.1 Theme 1: Stakeholder engagement as an art

This theme describes how, through the hidden curriculum, students learnt how to balance the interests and expectations of various internal (within the team or organization) and external stakeholders (such as partnering agencies, and beneficiaries of initiatives) in decision-making. This typically resulted from observing and experiencing collaborative dynamics in the workplace. Through these experiences, students developed skills in person-centric communication.

##### 3.2.1.1 Sub-theme 1: Collaborative dynamics

Firstly, students observed how public health practice often required working through differences in perspectives and agendas between stakeholders. This required multi-perspective taking, skilful communication and negotiation to align goals, reconcile differences, and resolve conflicts:

“*I witnessed the reconciliation of both differing yet valid perspectives through diplomatic negotiation and the willingness to adapt without compromising the integrity of our analysis.”* [ID1R2]

Secondly, the mentor-mentee relationship created teachable moments; these are further discussed below. We considered mentors as internal stakeholders, since they were involved in guiding interns in the project tasks assigned.

Many students felt they were given a place at the table despite being interns, as their mentors empowered them by granting adequate autonomy, role-modeling positive attributes, motivating them to improve through a nurturing approach:

“*When my turn came, I said something brief, factual, correct. One of the senior team members didn't respond to my summary. Instead, asked, ‘So what's your take on all this?' and ‘Why is it this way?'... I was being nudged to move beyond data collection and into meaning-making… It was also a subtle invitation to do more*.” [ID10R2]

Two students described the mentor-mentee experience as invalidating, particularly when mentors' interventions left them feeling inadequate. Despite this, both students extracted valuable lessons from these experiences. One of them recounted learning the importance of respecting organizational hierarchy following a critical incident:

“*My supervisor [was] extremely irritated… She said I should've gone through her; each member of the department has their own responsibility, and if someone does not respect their boundaries, it can easily cause troubles... I should've been aware of and sticked [sic] to who I can approach and who I cannot… If I were a full-time professional, not an intern, it would effectively close off opportunities for career advancement…”* [ID5R2]

Some students described help-seeking as a balancing act. This difficulty could stem from a self-imposed pressure to understand everything, especially within the initial weeks of the internship despite being new to the role, coupled with uncertainty about what to ask given the absence of a structured syllabus unlike in school, all while not wanting to burden others. Consequently, students learnt to discern how to navigate between self-directed learning and help-seeking:

“*No one explicitly said, ‘don't ask too many questions,'…I learnt to stagger my questions, to package them together in a concise way, and to show that I had tried other avenues first before asking.”* [ID10R2]

Thirdly, students observed how competing priorities in clinical settings sometimes deprioritised public health work. As such projects often required input from clinicians whose time was largely dedicated to patient consultations, one student reflected on how this affected stakeholder communication:

“*[The clinical professionals'] opinions were always valued and prioritized over those of the researchers, ensuring that our project truly met their needs… Yet, I observed frequent delays in their communication… [They] are often extremely busy [and] had little time to allocate for researchers…”* [ID5R1]

##### 3.2.1.2 Sub-theme 2: Person-centric communication

Students came to appreciate, often through observation and informal guidance from senior colleagues, the importance of effective framing. A student reflected how messaging had to be carefully tailored to the specific stakeholder being engaged:

“*When engaging policymakers, we were advised to highlight fiscal sustainability and implementation feasibility—key concerns for many decision-makers. When working with country teams, we focused more on service delivery gaps and community alignment. Every recommendation had to be tailored not just in content, but in tone.”* [ID1R2]

Through engagement with diverse stakeholder groups, including older populations, sex workers, and country-level teams, students developed cultural competence rarely addressed in the formal curriculum. They reflected on context-specific public health communication and navigating language barriers. A student recounted working on a global health project with a team in a neighboring country:

“*Language barriers… taught me to be more attuned to cultural and linguistic differences, and more importantly, to slow down, listen actively, and support team members in expressing their perspectives.”* [ID13R2]

Students' reflections on working with patients and vulnerable populations had a strong humanistic thrust, highlighting sensitivity, open-mindedness, and interpersonal skills as foundations for building trust with these groups. One student reflected on what it means to engage with a beneficiary on a fundamentally human level:

“*Having a personal touch as a health professional can go a long way in improving patient experience....”* [ID18R2]

#### 3.2.2 Theme 2: Effecting real change takes time

This theme describes how students realized that bringing about real change in public health was slow and tedious due to multi-level barriers, although change agency was evident in many work environments.

##### 3.2.2.1 Sub-theme 1: Organizational structure as a determinant of change

Students often discussed how structural inefficiencies and constraints, such as organizational bureaucracy, limited data access due to organizational policies, and institutional ethics application processes, hindered progress at work. Students also remarked how there seemed to be more administrative work than expected in public health. A student remarked:

“*…Solutions often remain confined to a single ward, or end on a pilot trial. Alignment needed to come from a wider range of stakeholders, from procurement to housekeeping, or from senior leadership to external vendors.”* [ID11R2]

Several students observed how organizational factors such as the inherent constraints of certain departmental roles and unrealistic performance targets shaped workplace behavior and attitudes, consequently undermining the pace of change. One student described how certain employees engaged in strategic manipulation of performance metrics to satisfy key performance indicator (KPI) requirements, a practice that drove the setting of progressively inflated targets for subsequent years, even as employees remained under sustained pressure to meet or exceed them:

“*Some KPIs are based on the previous year's standards which might be skewed due to workers ‘gaming the system'. But because it is officially reported that the organization hit the KPI… every year after they are expected to hit the same KPI or more, which might become unachievable such that ‘doing it the right way' would not be feasible due to the highly set KPI.”* [ID7R2]

Students described how public health practice tended to favor implementing incremental changes rather than novel solutions due to organizational-level considerations:

“*I observed how study findings were subtly shaped to align with ongoing institutional efforts, rather than being used to challenge assumptions or generate entirely new directions… Any novel solutions proposed were met by constraints such as limited manpower and financial resources.”* [ID4R2]

Although students acknowledged the ways in which organizational factors can impede change, several also recognized that the structural elements presenting barriers to rapid change serve a necessary function within organizations. They noted the value of interdisciplinary collaboration, cross-departmental interdependency in co-creating solutions, and the importance of institutional policies in providing an operational framework. A student remarked:

“*At [Organization F], adherence to regulations and guidelines for medical devices is a significant part of the process… involving extensive paperwork for traceability and audit. I have learned the importance of these policies and regulations for ensuring the safety, efficacy, and quality of medical devices.”* [ID14R2]

##### 3.2.2.2 Sub-theme 2: Broader-level barriers

Barriers to change in public health went beyond organizational factors and encompassed macro-level systemic challenges. As one student interning at a global health organization observed:

“*…While discussing and identifying the necessary actions is one thing, executing them is another. Many of the points discussed require time due to external factors such as political will, availability of resources, and the establishment of guidelines for outbreak management and response.”* [ID14R1]

##### 3.2.2.3 Sub-theme 3: Recipient-level barriers

The effectiveness of public health initiatives can also be hampered by barriers at the recipient level. Students witnessed first-hand how beneficiaries may not fully engage for a range of reasons, including fatigue and language barriers. A student shared how a beneficiary's deep-seated beliefs limited programme impact:

“*When a facilitator advised reducing fat intake, a senior quickly countered with a personal anecdote about a friend who ate fatty foods and remained healthy well into old age, [illustrating] how public health messaging is often filtered through deeply held personal beliefs and cultural norms.”* [ID4R2]

##### 3.2.2.4 Sub-theme 4: Agency despite constraints

Despite witnessing the challenges inherent to public health practice, students drew inspiration from colleagues and external stakeholders, who continued to drive change in the face of real-world constraints. In doing so, students observed how experienced practitioners did not regard challenges merely as problems to be resolved so much as conditions to be navigated while doing meaningful work:

“*Instead of waiting for change to happen from top-down… the team is constantly sourcing for vendors, and establishing partnerships... This system is built on people, and without determined and passionate individuals who want to drive change, there will always come a point where progress stagnates.”* [ID11R2]

#### 3.2.3 Theme 3: Developing personal effectiveness as a public health professional

This theme describes how the hidden curriculum of public health practice, conveyed through informal workplace experiences, shaped one's personal effectiveness, through resilience-building, managing personal-work values incongruence, self-evaluation of career fit, and broadening one's worldview.

##### 3.2.3.1 Sub-theme 1: Building resilience

Two key characteristics of resilience building—growth mindset and self-leadership—often developed through exposure to workplace adversity, were evident across many reflection scripts. Students described a greater sense of societal contribution instilled by the internship, embracing feedback as a vehicle for personal development, and intending to carry forward the lessons gained. Self-leadership was demonstrated through performing under pressure (for example, managing time amidst multiple tasks or remote work); taking ownership of work; developing adaptability, foresight, confidence, and overcoming discomfort or fear. One student commented:

“*This is the first time I am tasked with two ongoing projects, and it can be difficult to juggle the workload... I have been managing by prioritizing the more pressing project. Planning my work by setting goals and internal deadlines has also helped...”* [ID9R1]

##### 3.2.3.2 Sub-theme 2: Managing personal-work values incongruence

One student recognized the importance of delineating professional boundaries when the organizational mission gave rise to experiences that conflicted with their personal values, the latter shaped by their upbringing and sociocultural background:

“*I understood my role was to help [clients] in need and advocate for basic human rights, not to pass judgement... My work at the organization required me to confront my own beliefs about marriage, sex, and love, which were sometimes at odds with the perspectives of the [clients] and my colleagues. As a health professional, one must have a personal and professional balance, and serve all patients equally regardless of background.”* [ID18R2]

##### 3.2.3.3 Sub-theme 3: Self-evaluation of career fit

By experiencing the day-to-day realities of public health roles firsthand, students arrived at their own assessments of how well their roles aligned with their personal and career aspirations. While some found the experience fulfilling or a valuable addition to their professional portfolio, others concluded that the role was not well-suited to their longer-term career trajectory:

“*Coming into the internship, my impression was that public health professionals are often on the ground and directly interacting with the public. However, the reality I observed was quite different. A significant portion of the work…mostly happen [sic] in an office setting... I may not thrive in such an environment… The work does not align well with what I want in my career.”* [ID17R2]

##### 3.2.3.4 Sub-theme 4: Broadening of worldview

Several students reflected on how the internship had broadened their worldview. They reported developing a more grounded lens in public health, gaining a deeper appreciation of non-profit work and the roles of other healthcare professionals, and a greater awareness of their roles in society as budding professionals:

“*This exposure challenged my previous limited view... I learnt that effective public health practice is not about applying universal solutions, but about navigating nuance and responding with context-sensitive and realistic approaches.”* [ID1R2]

## 4 Discussion

This study explored the hidden curriculum in public health through undergraduate non-medical student interns, a perspective that has received limited attention in the literature. Students' reflections suggest Kolb's (35) experiential learning cycle manifested across their internships, which often collided with prior assumptions about the profession and society. It was through these tensions that the hidden curriculum became visible. Such reflection helped students revise their existing knowledge and assumptions, shaping their approach to future practice, moving from concrete experience, through reflection, to conceptualization and action.

Our findings elucidate undercurrents of power and hierarchy in public health practice. In high power-distance environments, students may find mentoring invalidating, learning whom they “can approach and who [they] cannot”, and that challenging authority could have severe consequences. Through such experiences, they internalized the notion that one should not challenge one's superiors. Interns' perception of help-seeking as a balancing act may stem from tension between a self-imposed expectation of independent learning and educational norms that had previously engendered dependency on authoritative figures for guidance (32).

Notwithstanding broader organizational dynamics, teams may develop microcultures that vary in how power distance is enacted, influencing team dynamics. Some students described the mentee-mentor experience as empowering, particularly when mentors made deliberate efforts to reduce power distance and foster open communication.

The deprioritisation of public health work in clinical settings parallels findings that power imbalance can hinder collaborative efforts in healthcare and affects team dynamics and decision-making (36). Generational differences in preference for low-power distance environments have been reported by others (36–38). How power dynamics evolve with time and influence the hidden curriculum in public health warrants further investigation.

The power dynamics lens extends to public health professional-client relationships. Students encountered tension between personal and work values when working with vulnerable groups, learning to suspend judgement and developing a greater sense of societal responsibility. This suspension, however, may obscure the underlying power asymmetry in such relationships, leaving students less attuned to how structural inequities, rather than individual attitudes alone, shape the vulnerabilities of the populations they serve (39).

A number of themes were consistent with hidden curriculum research in clinical disciplines, including humanism (9, 11, 18, 40), unprofessional behaviors (9, 11, 17), mentoring (9, 11, 18), cultural competence (17, 41), resilience-building (9, 17), personal-work values incongruence (11, 48), and self-evaluation of career fit (42).

Help-seeking as a balancing act was also suggested in another hidden curriculum study in health professions education (43). While that study examined the experiences of minoritised students, ours involved a multi-ethnic demographic, suggesting that balancing independent problem-solving and help-seeking may be an overarching developmental milestone in professional formation.

The unique scope of public health practice also surfaced themes absent from prior literature, such as effective framing, how effecting change takes time, organizational influences on workplace behavior, agency despite constraints, and broadening of worldview, suggesting that internships effectively extended academic learning. Whether these elements are salient to public health practice warrants further systematic review.

Stakeholder engagement, while not explicitly stated as a standalone competency in public health education frameworks (44, 45), is an essential cross-cutting skill in public health practice demanding multi-perspective taking and the ability to engage audiences beyond patients and clinicians (46). Although the formal curriculum may partly address this through public health communication courses, internships afforded richer, authentic contexts and opportunities for developing this skill. Through observing senior colleagues, students learnt the art of stakeholder engagement, including nuanced framing for engaging industry partners or the community, which often involves deep understanding of stakeholder contexts and motivations.

That real change takes time was a distinctive theme. While formal curricula emphasize effecting change through the teaching of relevant public health methods and contexts, the slow and often arduous process of doing so is not systematically covered. Concepts such as political will, resource constraints, and individual-level barriers to behavior change may be introduced formally, but learning is deepened when students witness first-hand why change in public health is rarely immediate, consistent with the experiential learning process described above. These align with Brown et al.'s (48) observations that certain aspects of the hidden curriculum are more effectively acquired through real-world practice, as students grasp the implications of broader- and recipient-level barriers to change during their internships.

Organizational structure as a determinant of change was another major sub-theme. While prior research found that structural elements such as organizational guidelines cultivated ethical values in clinical professions (9), our study suggested organizational policies functioned as a double-edged sword, necessary for institutional function but yet simultaneously hindering real change. Their influence on workplace behavior, such as gaming the system or favoring incremental changes over novel solutions, illustrated how corporate elements can be a necessary evil in public health practice. These sub-themes featured minimally in clinical hidden curriculum research but were otherwise highlighted in our study, likely reflecting the more pivotal role that organizational and policy structures play in public health.

The sub-themes “agency despite constraints” and “broadening of worldview” reflected students' capacity to make sense of their internship experiences within a larger professional and social context. Although not explicitly reported elsewhere, such differences in thematic framing may reflect the lack of a harmonized hidden curriculum definition (9) rather than their absence in experience, as illustrative quotes in the wider literature suggest these elements were present but unreported as thematic foci (11, 41, 47).

Contrary to existing literature (13, 28, 29, 43, 48), themes of race, gender, and dress code were not identified in our study, possibly because the extant public health hidden curriculum literature is mainly limited to opinion pieces and textbook analyses (28–31). Moreover, the broader empirical hidden curriculum research remains largely situated in non-Asian contexts (8, 11, 13, 47, 48), tending to reflect dominant values of those societies. Crucially, students' framing and selection of experiences disclosed may have been influenced by our study's setting in a multicultural Southeast Asian society that prioritizes social harmony over divisive differences (49), coupled with educational cultural norms around power distance in student-teacher relationships (32, 50). Participants were not restricted to selected subgroups, which may further account for such contrasts.

### 4.1 Implications on practice

Our study findings affirm the pedagogical value of internships and academic curricula in introducing the hidden curriculum in public health by exposing students to tensions between theory and practice. While we acknowledge Brown et al.'s (48) observations that not all negative aspects of the hidden curriculum can be effectively addressed from the classroom educational practice standpoint, especially those rooted in macro-level factors, we are also of the view that not every negative aspect should necessarily be mitigated. Echoing Ng et al. (51), protecting students from such tensions and the negative aspects of the hidden curriculum would risk short-changing them of valuable learning opportunities. Rather than eliminating exposure to the hidden curriculum, educators can better prepare students to confront it.

At the programme level, learning activities fostering perspective-taking skills and exposure to diverse worldviews and cultures (52, 53) should be intentionally integrated into public health curricula to equip students with foundational skills and bridge the expectation-reality gap as they enter the workforce. Reflective practice, a key characteristic of competent professionals (54), entails “examining assumptions, power relations, and how these shape practice” (55). Critical reflection could therefore be embedded across courses with experiential learning components to prime learners for the hidden curriculum before encountering it directly. At the faculty/staff level, positive role-modeling and supportive mentorship can inculcate the right attitudes, behaviors, and values embodied in public health, while also helping students process academic and workplace experiences (56). For instance, since clinicians' deprioritisation of public health work may reflect broader systemic issues, mentors could guide students in engaging specific stakeholders more effectively.

### 4.2 Limitations and directions for future research

Our findings' transferability may be limited by the small, predominantly female (69%), single institution sample. Although ungraded, reflections were submitted as part of formal coursework. This may have introduced some social desirability bias in how experiences were framed or disclosed. Reflections were also collected over one semester per cohort, precluding the systematic examination of how students' perspectives evolved over time; longitudinal studies would help address this.

To our knowledge, however, this is the first empirical study to examine the hidden curriculum in public health, and one situated within a multi-ethnic Asian context, enriching a predominantly Western-centric, clinically focused literature. Larger, more diverse, and non-institutionally-linked samples within and beyond Asia would help establish whether these themes hold in other cultural settings and mitigate potential social desirability bias. Research on experienced practitioners' perspectives would complement this student-focused study for a fuller picture of how the hidden curriculum shapes professional formation in public health.

## 5 Conclusion

This study explored the hidden curriculum in public health through undergraduate non-medical public health interns, introducing perspectives from a multi-ethnic Asian student population in Singapore. Both positive and negative aspects of the hidden curriculum were highlighted. Future research involving larger, more diverse, and non-institutionally-linked samples, practitioners' perspectives, and longitudinal follow-up would better characterize the hidden curriculum in public health and inform future professional training.

## Data Availability

The data supporting the findings of this study are available on request to the corresponding author.
