# Supplementary material for: Exploring the hidden curriculum in public health: a qualitative study
Source: Front Public Health. 2026 Oct 22;14:1945036. doi: 10.3389/fpubh.2026.1945036 (PMC13736580; doi:10.3389/fpubh.2026.1945036)
Supplement: Supplementary file 1 [file Table_1.docx]

**Supplementary Materials**

**Table S1.** Themes, subthemes, and illustrative quotes

| **Themes** | **Level 1 subthemes** | **Level 2 subthemes** | **Level 3 subthemes** | **Illustrative quotes** |
| --- | --- | --- | --- | --- |
| Stakeholder engagement as an art | Collaborative dynamics | Working through differences |  | *“I witnessed the reconciliation of both differing yet valid perspectives through diplomatic negotiation and the willingness to adapt without compromising the integrity of our analysis.”* [ID1R2] |
|  |  | Mentoring | Mentoring as empowering | *“When my turn came, I said something brief, factual, correct. One of the senior team members didn’t respond to my summary. Instead, asked, ‘So what’s your take on all this?’ and ‘Why is it this way?’... I was being nudged to move beyond data collection and into meaning-making… It was also a subtle invitation to do more.”* [ID10R2] |
|  |  |  | Mentoring as invalidating | *“My supervisor [was] extremely irritated… She said I should’ve gone through her; each member of the department has their own responsibility, and if someone does not respect their boundaries, it can easily cause troubles... I should’ve been aware of and sticked [sic] to who I can approach and who I cannot… If I were a full-time professional, not an intern, it would effectively close off opportunities for career advancement…”* [ID5R2] |
|  |  |  | Help-seeking as a balancing act | *“No one explicitly said, ‘don’t ask too many questions,’ …I learnt to stagger my questions, to package them together in a concise way, and to show that I had tried other avenues first before asking.”* [ID10R2] |
|  |  | Competing priorities in clinical settings |  | *“[The clinical professionals’] opinions were always valued and prioritized over those of the researchers, ensuring that our project truly met their needs… Yet, I observed frequent delays in their communication… [They] are often extremely busy [and] had little time to allocate for researchers…”* [ID5R1] |
|  | Person-centric communication | Framing |  | *“When engaging policymakers, we were advised to highlight fiscal sustainability and implementation feasibility—key concerns for many decision-makers. When working with country teams, we focused more on service delivery gaps and community alignment. Every recommendation had to be tailored not just in content, but in tone.”* [ID1R2] |
|  |  | Cultural competence |  | *“Language barriers… taught me to be more attuned to cultural and linguistic differences, and more importantly, to slow down, listen actively, and support team members in expressing their perspectives.”* [ID13R2] |
|  |  | Humanism |  | *“Having a personal touch as a health professional can go a long way in improving patient experience....”* [ID18R2] |
| Effecting real change takes time | Organisational structure as a determinant of change | Impedes meaningful change | Structural inefficiencies and constraints | *“…Solutions often remain confined to a single ward, or end on a pilot trial. Alignment needed to come from a wider range of stakeholders, from procurement to housekeeping, or from senior leadership to external vendors.”* [ID11R2] |
|  |  |  |  |  |
|  |  |  |  |  |
|  |  |  | Influence on workplace behaviour and attitudes | *“Some KPIs are based on the previous year’s standards which might be skewed due to workers ‘gaming the system’. But because it is officially reported that the organisation hit the KPI… every year after they are expected to hit the same KPI or more, which might become unachievable such that ‘doing it the right way’ would not be feasible due to the highly set KPI.”* [ID7R2] |
|  |  |  | Implementing incremental changes over novel solutions | *“I observed how study findings were subtly shaped to align with ongoing institutional efforts, rather than being used to challenge assumptions or generate entirely new directions… Any novel solutions proposed were met by constraints such as limited manpower and financial resources.”* [ID4R2] |
|  |  | A necessity |  | *“At [Organisation F], adherence to regulations and guidelines for medical devices is a significant part of the process… involving extensive paperwork for traceability and audit. I have learned the importance of these policies and regulations for ensuring the safety, efficacy, and quality of medical devices.”* [ID14R2] |
|  | Broader-level barriers |  |  | *“…While discussing and identifying the necessary actions is one thing, executing them is another. Many of the points discussed require time due to external factors such as political will, availability of resources, and the establishment of guidelines for outbreak management and response.”* [ID14R1] |
|  | Recipient-level barriers |  |  | *“When a facilitator advised reducing fat intake, a senior quickly countered with a personal anecdote about a friend who ate fatty foods and remained healthy well into old age, [illustrating] how public health messaging is often filtered through deeply held personal beliefs and cultural norms.”* [ID4R2] |
|  | Agency despite constraints |  |  | *“Instead of waiting for change to happen from top-down… the team is constantly sourcing for vendors, and establishing partnerships... This system is built on people, and without determined and passionate individuals who want to drive change, there will always come a point where progress stagnates.”* [ID11R2] |
| Developing personal effectiveness as a public health professional | Building resilience |  | Self-leadership | *“This is the first time I am tasked with two ongoing projects, and it can be difficult to juggle the workload... I have been managing by prioritizing the more pressing project. Planning my work by setting goals and internal deadlines has also helped...”* [ID9R1] |
|  |  |  | Growth mindset | *“Through these experiences… I feel empowered to be able to think more deeply about how we can build better healthcare infrastructure... I hope to be able to hold these ideas close to me amidst my journey to being a more effective healthcare*  *worker [and] promote unwritten rules that benefit society while inspiring change for those unwritten rules that hold us back.”* [ID7R2] |
|  | Managing personal-work values incongruence |  |  | *“I understood my role was to help [clients] in need and advocate for basic human rights, not to pass judgement... My work at the organisation required me to confront my own beliefs about marriage, sex, and love, which were sometimes at odds with the perspectives of the [clients] and my colleagues. As a health professional, one must have a personal and professional balance, and serve all patients equally regardless of background.”* [ID18R2] |
|  | Self-evaluation of career fit |  |  | *“Coming into the internship, my impression was that public health professionals are often on the ground and directly interacting with the public. However, the reality I observed was quite different. A significant portion of the work…mostly happen [sic] in an office setting... I may not thrive in such an environment… The work does not align well with what I want in my career.”* [ID17R2] |
|  | Broadening of worldview |  |  | *“This exposure challenged my previous limited view... I learnt that effective public health practice is not about applying universal solutions, but about navigating nuance and responding with context-sensitive and realistic approaches.”* [ID1R2] |

**S2	Reflection prompts**

The reflection prompts used to guide interns in their written reflections which in turn formed the primary data source for this study are provided in italics below.

*Every intern’s experience is unique and personal. Use the guiding prompts to help you reflect on and self-assess your internship experience. For each entry, please keep to the word limit stipulated for each reflection.*

*Reflection 1 (max 500 words)*

*What was one difficulty you faced? Why was it a dilemma? What were some options you considered doing to address the problem? How did you eventually overcome it? This challenge may refer to anything within the context of your internship.*

*Reflection 2 (max 1500 words)*

*The “hidden curriculum” is the set of influences on one’s development as a public health professional that is not explicitly taught. It is transmitted through interpersonal interactions in practice settings, through positive or negative role model behaviours, and through the culture and hierarchy of public health.*

*Examples of what might be learnt from the hidden curriculum include the unspoken “rules to survive” at a particular institution, the accepted manner of interacting with internal and external stakeholders, attitudes toward and treatment of difficult or marginalised persons, ethical dilemmas, and choices about personal/professional balance.*

*The hidden curriculum influences the values, roles, and identity a public health professional develops over the course of training. The hidden curriculum is a strong socialising force, and its influence can be positive, negative, or mixed.*

*In no more than 1500 words, discuss about the hidden curriculum as you have observed it during your internship experience. Consider the extract above and the following prompts to help you understand what you need to reflect about:*

- *What were some aspects that you have observed that were different from, or not covered in, your public health-related academic experience? In other words, what have you observed about how different is “what we say we do” from “what we actually do” in public health practice?*
- *What were some “unwritten rules” that you observed? How have these affected you positively and/or negatively?*

*We suggest that you start out with a personal anecdote or story that epitomises some aspect of the hidden curriculum and use this anecdote as a starting point for your reflection.*
